# RUNX1 mediates the therapeutic effects of mesenchymal stem cells‐derived microparticles in acute respiratory distress syndrome

**DOI:** 10.1002/ctm2.1455

**Published:** 2023-11-13

**Authors:** Dan Predescu, Shanshan Qin, Vasilios Stefanis, Brandon Carman, Balaji Ganesh, Kanika Sharma, Babak Mokhlesi, Sanda Predescu

**Affiliations:** ^1^ Department of Internal Medicine Rush University Medical Center Chicago Illinois USA; ^2^ Rush University Medical Center Chicago Illinois USA; ^3^ Department of Pharmacology University of Illinois Chicago Chicago Illinois USA

Dear Editor,

Recently, we reported the presence in the systemic circulation of mesenchymal stem cell (MSC)‐derived microparticles (MPs) containing an isoform of the transcription factor RUNX1 (RUNX1*^p66^*), which is highly associated with improved survival of acute respiratory distress syndrome (ARDS) patients.^1^ In this study, we aimed to characterise the MPs‐containing RUNX1*^p66^* and compare their therapeutic effects with the parent MSCs.

Disruption of inter‐endothelial junctions (IEJs) is a main pathology of ARDS.^1^, ^2^ The beneficial effects of MPs versus parent MSCs on IEJs integrity were evaluated in lipopolysaccharide (LPS)‐injured pulmonary endothelial cells (EC_LPS_). MPs were isolated from the growth media of parent MSCs at day 7 [MPs(d7)], when the MPs express RUNX1*^p66^* and at days 5 and 8 [MPs(d5), MPs(d8)], when the MPs are RUNX1*^p66^*‐negative (Figure S1A). The MPs were characterised as per ISEV guidelines^3^ (Figure S1B–I). Untreated ECs (EC_Ctrl_), EC_LPS_ and the MSCs‐ and MPs‐treated EC_LPS_ were subjected to Z0‐1/Alexa Fluor 594 immunostaining (Figure 1A–G). The surface area of the intercellular gaps was used to measure the IEJs integrity/repair induced by MSCs and MPs treatment. MPs(d7) decreased by 2.5‐fold the surface of intercellular gaps, while MSCs, MPs(d5), and MPs(d8), by only 1.3‐fold (Figure 1H). Note, the apparent sealing process of the IEJs in MPs(d7)‐treated EC_LPS_, yellow box, and the higher magnification (Figure 1C,G). Comparable results were obtained for VE‐cadherin/Alexa Fluor 594 immunostaining (Figure S2A–G). The proliferative effects of MPs and MSCs on EC_LPS_ were evaluated by the EdU assay (Figure 1I–L), and Ki67 immunostaining (Figure S2H–M). A 1.8‐fold increase in proliferation of MPs(d7)‐treated EC_LPS_ versus EC_LPS_, which is 1.3‐fold greater than the MSCs and MPs(d5)‐treated EC_LPS_, was recorded 48 h post‐treatment (Figures 1 M and S2N). Thus, the MPs(d7) shows greater efficacy in improving junctional integrity and stimulating EC_LPS_ proliferation compared to parent MSCs. The beneficial effects of MPs and MSCs on EC_LPS_ show no significant sex differences. Given the direct contact MSCs/ECs monolayer, the possibility of interference with the proliferative cell counting was addressed by electron microscopy and PKH67 labelling of MSCs (Figure S3).

**FIGURE 1:**
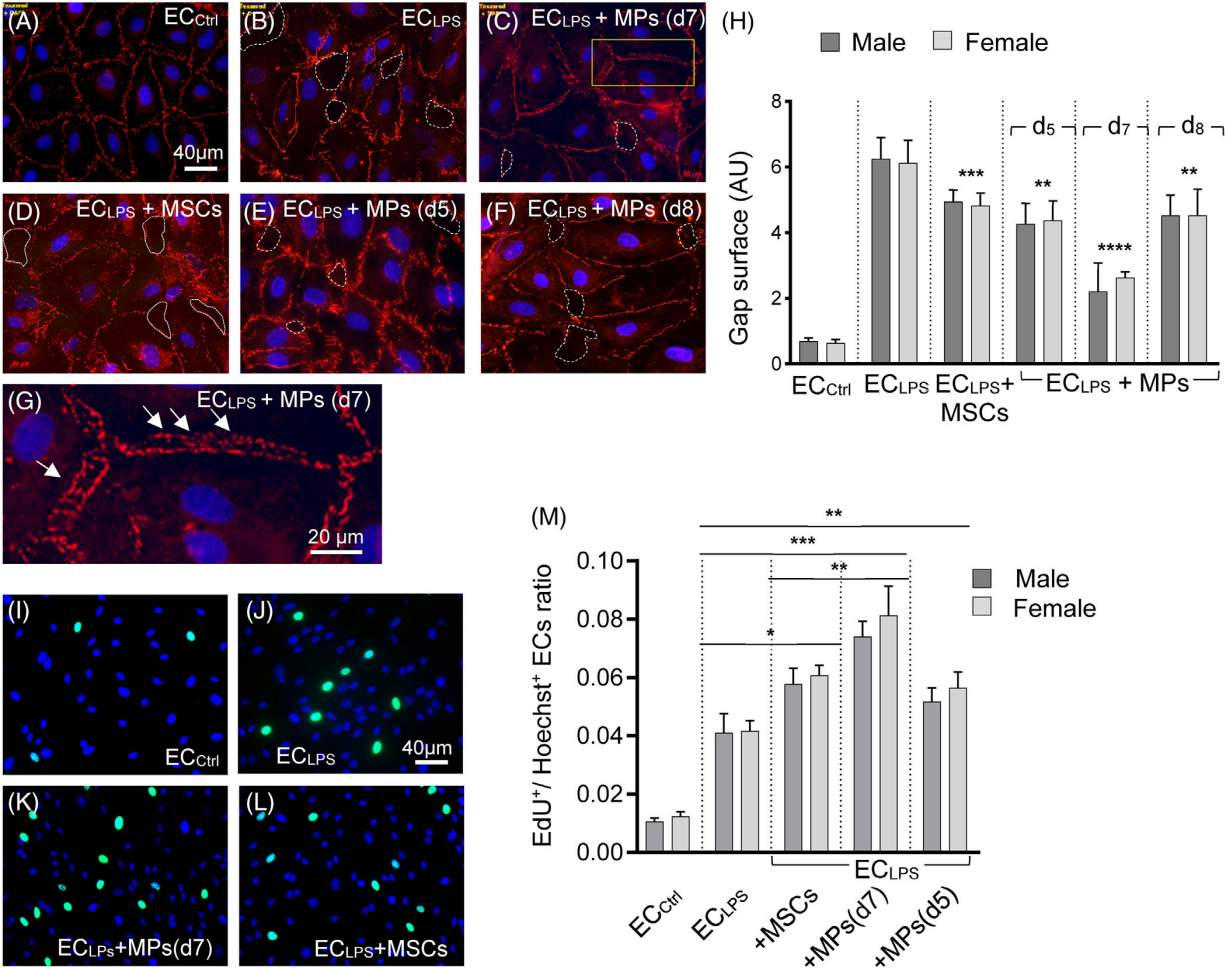
MPs‐immunoreactive to RUNX1p66 show greater efficacy in improving the IEJ integrity and stimulating human pulmonary endothelial cell (EC) proliferation compared to parental MSCs. Representative Z0‐1/Alexa Fluor 594 immunostaining of human lung microvascular ECs, male (M) and female (F, shown) donors Lonza (Walkersville, Inc., MD) as follows: (A) ECCtrl, (B) ECLPS (1 μg/mL LPS, 6 h), (C) ECLPS exposed to 10 μg MPs(d7), (D) ECLPS exposed to 105 MSCs, (E) ECLPS exposed to 10 μg MPs(d5) and (F) ECLPS exposed to 10 μg MPs(d8), all for 30 h. The amounts of MPs and MSCs employed are equivalent and fall in the range of previously published in vitro studies.^9^ As expected, ECLPS show altered cell morphology such as cell rounding, shrinking, and intercellular gap formation, and Z0‐1 subcellular re‐distribution. The intercellular gaps were depicted with dashed white lines. The yellow box in C, and the higher magnification in (G) illustrate the apparent sealing process of the IEJs in MPs(d7)‐treated ECLPS and the proximity between the two ECs plasma membranes (arrows). Images were acquired with a Zeiss AxioImager M1 motorised microscope equipped with an AxioCam ICc1 R3 RGB colour digital camera. Bars: 40 μm (A–F) and 20 μm (G). (H) Quantification of the surface of the intercellular gaps (NIH ImageJ software). Data are shown as the average gap surface per 50 high‐power fields of view. ***p < .0001 (ECLPS + MSCs vs. ECLPS); **p < .0002 ([ECLPS + MPs (d5, d8)] vs. ECLPS: ****p < .0001 [ECLPS + MPs (d7) vs. ECLPS]. The averages of gap surface for EC‐M and EC‐F were used for statistical analyses. AU—arbitrary units. The Student's *t*‐test (two‐tailed, unpaired *t*‐tests) was used to compare samples and their controls for all experiments (GraphPad Prizm 8.2.1 software). (I) Female ECCtrl, (J) ECLPS, (K) ECLPS exposed to 10 μg MPs(d7) immunoreactive to RUNX1p66, and (L) ECLPS exposed to 105 parent MSCs were subjected to EdU proliferation assay. Bar 40 μm (A—D) (M) Quantitative data (Cell Profiler software) are shown as EdU+/Hoechst+ ECs ratio. *p < .002 (ECLPS+MSCs vs. ECLPS); **p < .0003 (ECLPS+MPs vs. ECLPS: ***p < .009 (ECLPS+MPs vs. ECLPS +MSCs). The averages EdU+/Hoechst+ ratios for EC‐M and EC‐F, per 50 high‐power fields of view were used for statistical analyses. Bar: 40 μm (I—L). All values are mean ± SD. *n* = 3 independent experiments performed in triplicates, using at least three different MP preparations isolated from the growth media of cultured human bone marrow derived MSCs, at different time points in culture. MSCs, donated by a 22‐year‐old male, were from the Institute of Regenerative Medicine, Texas A&M Health Science Center (Temple, TX) and used at passage 4−5.

The beneficial effects of MPs and MSCs in vivo were evaluated in LPS‐injected mice by measurements of blood oxygenation (SpO_2_; Figure 2A). At 96 h, the LPS‐injected mice treated with MSCs or MPs(d7), showed similar SpO_2_ values, only 6.2% lower than controls, while the non‐treated LPS‐injected mice were still hypoxemic, with 16% lower SpO_2_ values than controls. Lung histology and morphometry indicated comparable therapeutic effects of MPs(d7) and MSCs in reducing the perivascular cuffing (pvc) and the inflammatory cells, compared to LPS‐injected mice, which showed large pvc and inflammatory cells infiltrates (Figure 2D,E vs. C). No pvc and no inflammatory cells were detected in controls (Figure 2B). A five‐fold reduction in pvc, and a decrease of inflammatory cells by 2.2‐fold in the blood vessel lumen and 3.3‐fold in the pvc, all compared to LPS‐injected mice without treatment were recorded (Figure 2H,I). MPs(d5, d8) reduced the pvc area by only 3.2‐fold, the number of inflammatory cells by 1.45‐fold in the lumen, and 1.3‐fold in the pvc (Figure 2F–I). The complexity of lung environment and cell diversity may explain the similar therapeutic effects of MPs and parent MSCs on injured ECs in vivo. No beneficial effects of MPs and MSCs treatment on the hyperproliferative bronchial epithelium or fibroproliferation in the LPS‐injected mice have been detected (Figure S4).

**FIGURE 2:**
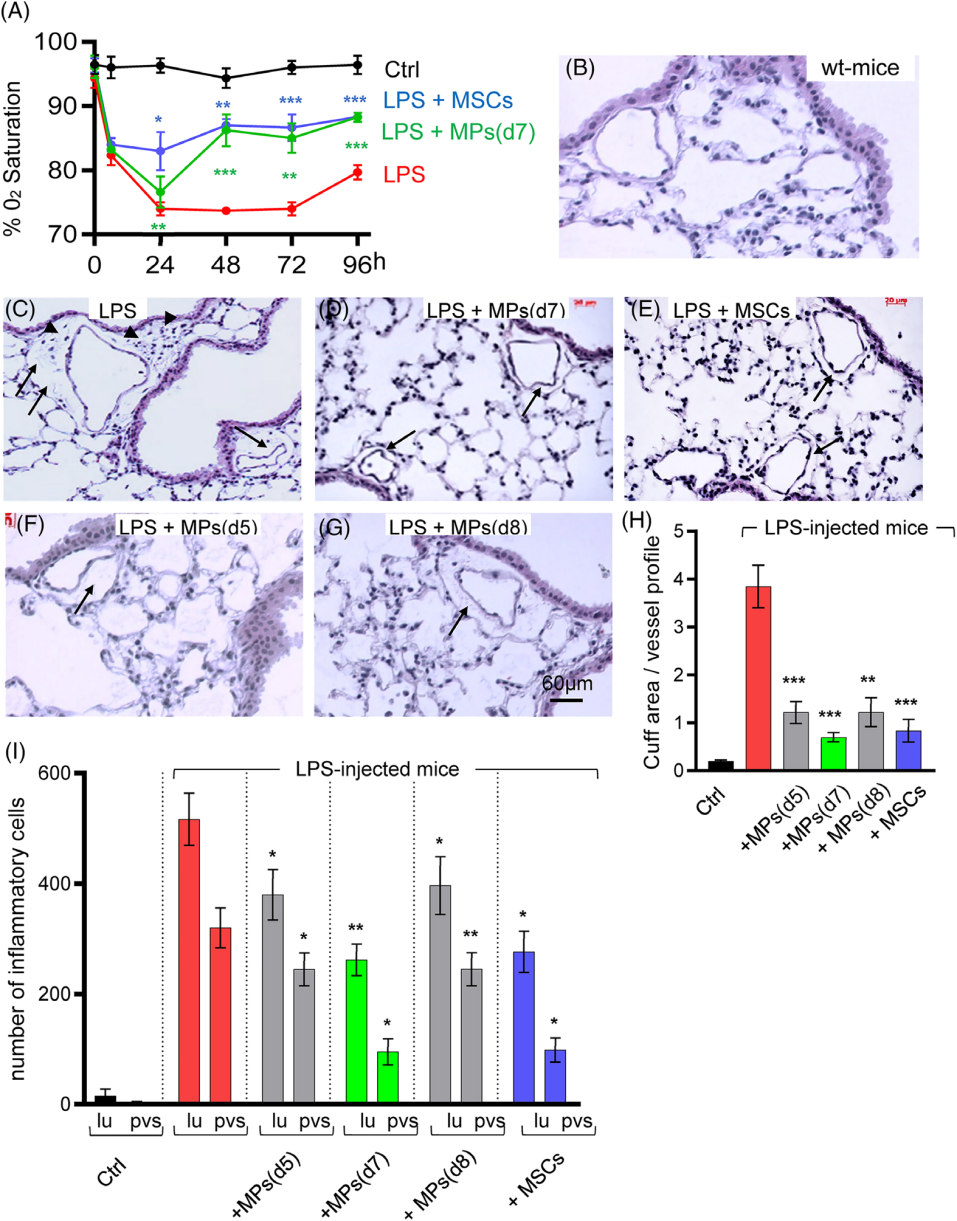
MPs‐immunoreactive to RUNX1p66 and parent MSCs equally restore blood oxygen levels (SpO_2_) in LPS‐injected mice. (A) Blood oxygen levels in mice injected i.p. with a sub‐lethal dose of 8 mg/kg LPS, followed by treatment with MPs (36 μg) or parental MSCs (2 × 105) in 100 μL PBS, at 6 h post‐LPS injection. A number of 2 × 105 cultured MSCs release on average 36–40 μg MPs protein, strong indication that the MPs and MSCs amounts used are equivalent. In addition, these MPs and MSC amounts fall in the range of previously published in vivo studies.^10^ The SpO_2_ readings at different time points as indicated were done for 3 min for each mouse. No statistically significant sex differences were detected. Sex‐aggregated data are shown. *n* = 3 independent experiments using 40 mice, 20 males and 20 females, 6−7 mice/group. Values mean ± SD. *p < .04; **p < .005; ***p < .007 vs. control mice. (B–G) Representative H&E staining of 4% paraformaldehyde‐fixed, paraffin‐embedded lung tissue sections of (B) wt‐mice, (C) LPS‐injected mice, (D) LPS‐injected mice treated with MPs(d7), (E) MSCs‐treated mice, (F) LPS‐injected mice treated with MPs(d5), and (G) LPS‐injected mice treated with MPs(d8), at 6 h post‐LPS injection. Arrows in (C–G) indicate the perivascular cuffs (pvc) and the arrowheads in (C) indicate the inflammatory cell infiltrates. Bar: 60 μm (B–G). (H) Quantification of pvc area per vessel profile. ***p < .003 MPs(d5) and **p < .0011 MPs(d8) vs. LPS‐injected mice; ***p < .0003 MPs(d7) and MSCs vs. LPS‐injected mice. (I) Quantification of the number of inflammatory cells in the lumen (lu) and perivascular space (pvs) in wt‐mice, LPS‐injected mice and LPS‐injected mice treated with MPs(d5), MPs(d7), MPs(d8) and MSCs. *p < .04 MPs (d5, lu, pvs), MPs (d8, lu) and MSCs (lu) vs. LPS‐injected mice (lu); **p < .001 MPs (d7, lu), MPs (d8, pvs); ***p < .0008 MPs (d7, pvs); MSCs (pvs) vs. LPS‐injected mice (pvs). All values are mean ± SD. *n* = 6 mice (3 males/3 females in three independent experiments, with three different MP preparations). Five to six stained lung sections per mouse were analysed. The Student's *t*‐test (two‐tailed, unpaired *t*‐tests) was used to compare samples and their controls (GraphPad Prizm 8.2.1 software).

As RUNX1 stability/transcriptional activity is regulated by posttranslational modifications, we suspected that RUNX1*^p66^* may be glycosylated (*O*‐GlcNAc), modification that accounts for preserving RUNX1 stability.^4^ The MPs from the blood of ARDS patients comprise several O‐GlcNAc proteins (Figure 3A, a1–a3), one of which is RUNX1*^p66^* as confirmed by WB with RUNX1 antibody (Figure 3A). No *O*‐GlcNAc proteins were detected in controls MPs lysates (Figure 3A, a4). To relate the EC_LPS_ injury to RUNX1 phosphorylation, we examined two potential sites for phosphorylation—Ser^249^ and Ser^276^. A decrease in RUNX1 phosphorylation, more evident at Ser^276^ compared to Ser^249^, 2 h post‐LPS exposure, and more obvious when normalised to total RUNX1 protein, was detected. Total RUNX1 was two‐fold increased 2 h post‐LPS exposure (Figure 3B), consistent with reports of RUNX1 up‐regulation in LPS‐injured lungs.^5^ Actin was not changed. The Phospho/Total RUNX1 ratio indicated a 1.5‐fold decrease in Ser^249^ and greater than four‐fold decrease in P‐Ser^276^ in EC_LPS_ versus EC_Ctrl_ (Figure 3C), consistent with a reduced level of transcriptionally active RUNX1. Thus, the MSCs‐derived MPs bearing the transcriptionally inactive *O*‐GlcNAc RUNX1*^p66^* protect it from proteasome‐mediated degradation and transfer it to injured EC_LPS_, leading to a recovery process. As RUNX1 degradation is mediated by the ubiquitin–proteasome pathway, ECs were depleted of Stub1, the E3‐ubiquitin ligase that promotes RUNX1 ubiquitination/degradation (Figure 3D).^6^ RUNX1 stability in Stub1‐depleted ECs was investigated in the presence of cycloheximide (Chx). Stub1 depletion resulted in greater than three‐fold RUNX1 accumulation (Figure 3E,F), while actin was two‐fold decreased (Figure 3E,G). RUNX1 ubiquitination/proteasome degradation was further documented in ECs grown in the presence of the proteasome inhibitor MG132 and MPs(d7)‐treated EC_LPS_ (Figure S5A–C,E).

**FIGURE 3:**
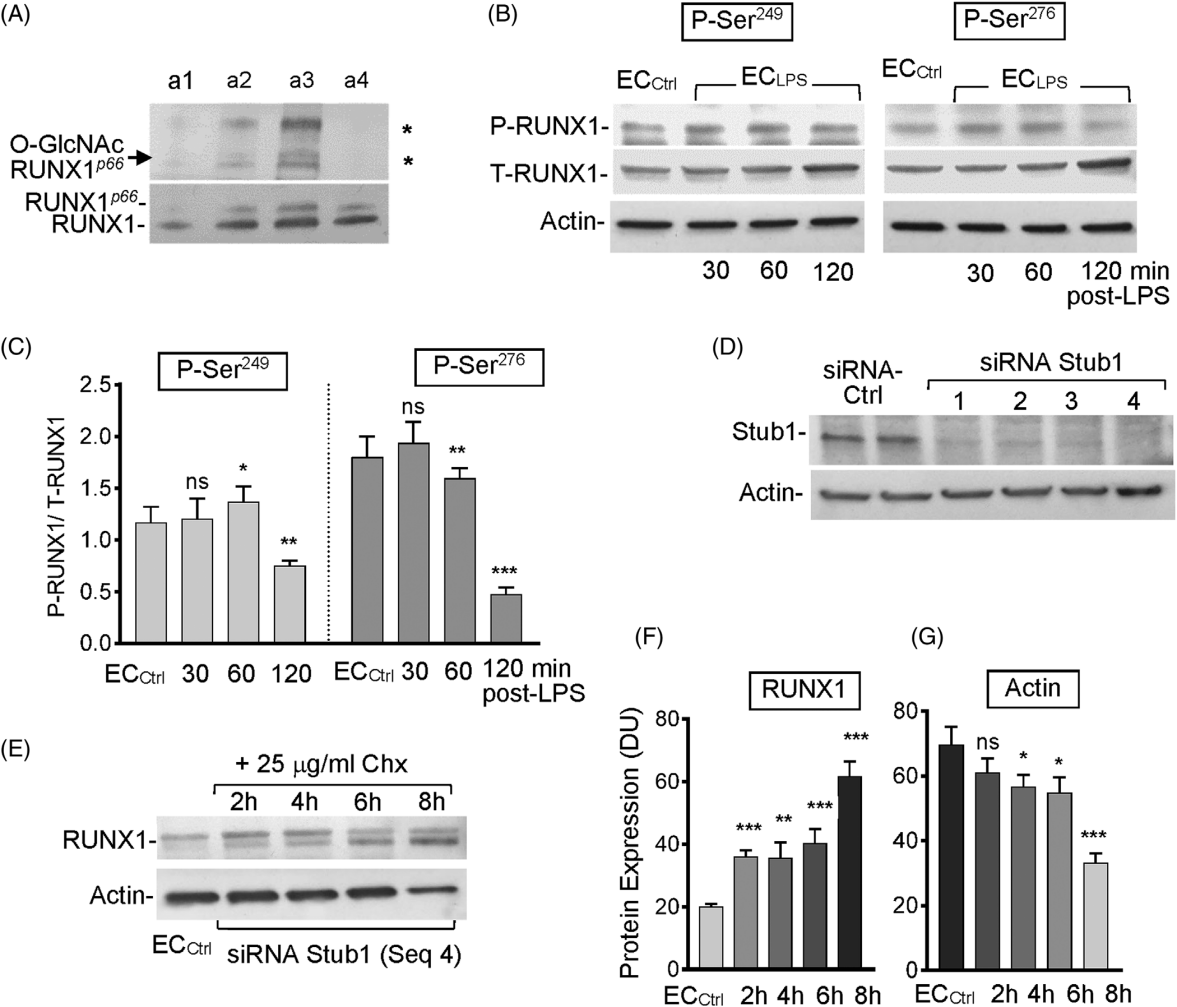
(A) RUNX1p66 is an O‐GlcNAc isoform. (A) Three different dilutions of a MPARDS lysate (a1, a2, a3) labelled with the Click‐iT™ O‐GlcNAc Enzymatic Labeling System [the permissive mutant  β‐1,4, galactosyl transferase which transfers azido‐modified galactose from UDP‐GalNAz to O‐GlcAc residues on the target proteins)] and the lysate of MPARDS subjected to the same procedure, without the addition of the Gal‐T1 enzyme (a4) were analysed by Sodium dodecyl‐sulfate polyacrylamide gel electrophoresis (SDS‐PAGE) and WB with Runx1 Ab. The lower panel shows the WB analysis of the same membrane using RUNX1 antibody. Four RUNX1p66‐immunoreactive MPARDS lysates were used. (B). Lysates of ECCtrl and ECLPS, 70 μg/lane, were analysed by WB for phosphorylation of RUNX1 (P‐RUNX1) at Ser249 and Ser276 (upper panels), for total (T)‐RUNX1 protein expression (middle panels) and actin, as a loading control (lower panels), using specific antibodies. *n* = 3 independent experiments. (C) Quantification of RUNX1 phosphorylation expressed as the ratio P‐RUNX1/T‐RUNX1; *n* = 3 independent experiments. *p < .01; **p < .02; ***p < .0004 vs. ECCtrl. Values are mean ± SD. (D) ECs were transfected with the human Stub1 SMARTPool siRNA (four individual siRNA RUNX1 sequences) and two siRNA control sequences. Actin was used as a loading control. (E) Cell lysates of ECCtrl and Stub1‐depleted cells cultured in the presence of 25 μg/mL cycloheximide (Chx; protein synthesis inhibitor), for 2 h, 4 h, 6 and 8 h as indicated, were subjected to SDS‐PAGE, and immunoblotting for RUNX1 and actin. On‐TARGETplus human Stub1 siRNA sequence UGGAAGAGUGCCAGCGAAA has been used for this experiment. Two ON‐TARGET*plus* Non‐targeting Control siRNA sequences (siRNA#1: UGGUUUACAUGUCGACUAA; siRNA#2: UGGUUUACAUGUUGUGUGA) served as negative controls. (F,G) Densitometric analyses of RUNX1 (left) and actin (right) immunoreactivity on X‐ray films, obtained in three independent experiments shown in Figure 4E. *p < .05; **p < .016; *** < .0005 vs. EC_Ctrl_. Values are mean ± SD.

MPs(d5) and MPs(d8) not‐immunoreactive to RUNX1*^p66^* support minimal cell proliferation suggesting the involvement of the lower Mr RUNX1 isoform. We performed RUNX1 loss‐of‐function studies, using siRNA (Figure 4A). RUNX1 was efficiently knocked down 72 h post‐transfection by comparison to EC_Ctrl_ or ECs treated with the siRNA_Ctrl_. Three days later, the number of cells was reduced by more than 16% compared to controls (Figure 4B), with no evidence of cell death. While other protective factors cannot be ruled out, the data suggest that RUNX1*^p66^* augments the proliferative effects RUNX1 on EC_LPS_. As RUNX1 is a target of Cdk6 and it regulates the time ECs spend in the G_1_ phase of the cell cycle,^7^ we examined the expression of Cdk6, a G_1_‐specific regulator, and of ccnd2, a G_1_/S specific cyclin (Figure 4C). WB and densitometry indicated a 2.2‐fold greater Cdk6 and 1.3‐fold greater ccnd2 expression in EC_LPS_ exposed to MPs(d7) compared to EC_Ctrl_ (Figure 4D). Thus, MPs(d7)‐EC_LPS_ interaction increases cell proliferation by upregulation of both components of the Cdk6−ccnd2 kinase complex. Finally, we show that the expression of RUNX1*^p66^* can be modulated in MSCs and their derived MPs, suggesting that the MPs can be engineered to enhance their efficacy (Figure S5E,F).

**FIGURE 4:**
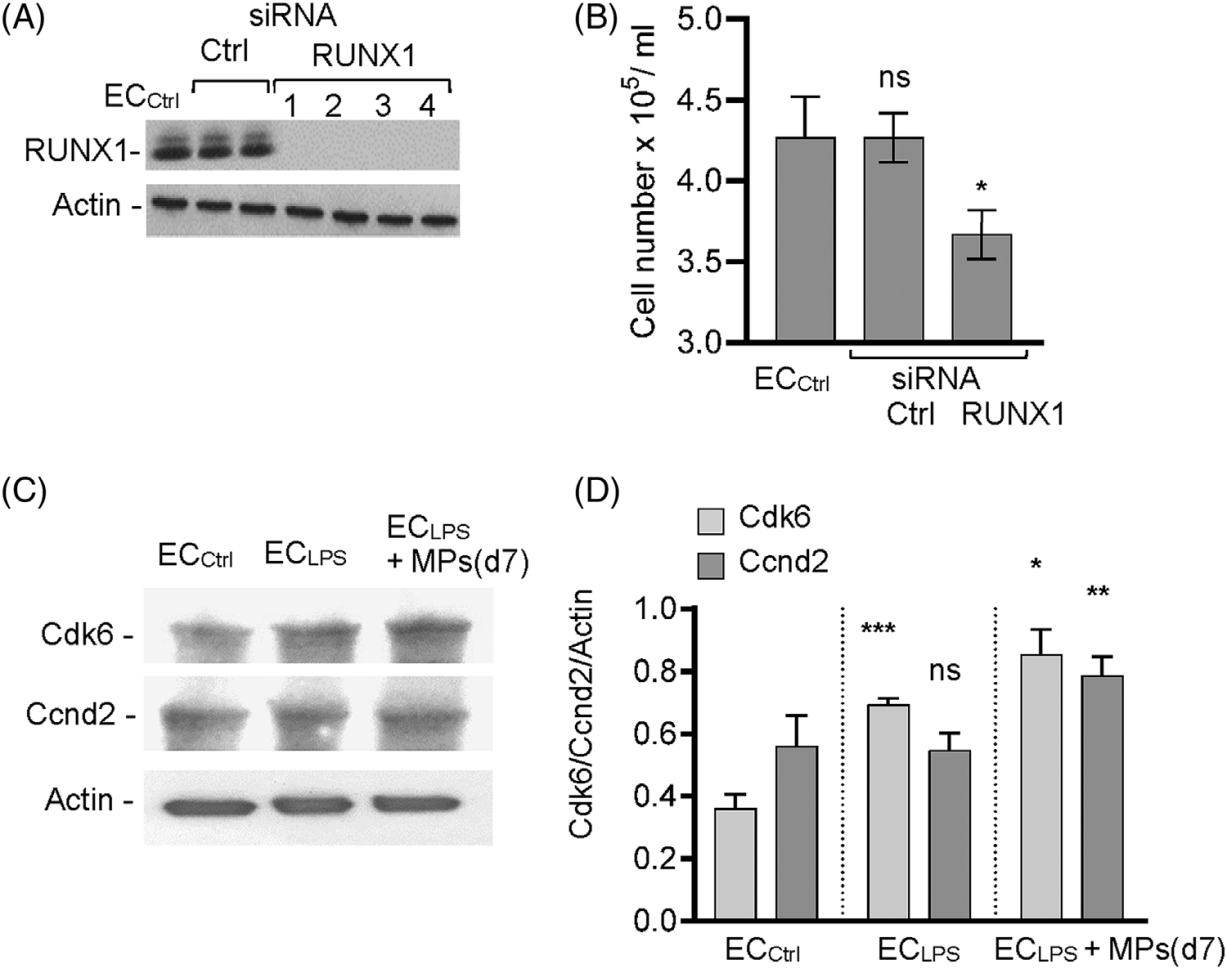
RUNX1p66 augments the proliferative effects of RUNX1. (A) ECs were transfected with siRNACtrl and RUNX1 SMARTpool Runx1 (four individual siRNA RUNX1 sequences) and analysed by WB with RUNX1 antibody. ECs were counted 3 days later. (B) The number of RUNX1 siRNA transfected cells is the average of all four siRNA sequences of three independent experiments (triplicate wells each). Active caspase‐3 staining showed no evidence of cell death in Runx1 siRNA transfected ECs, 72 h post‐transfection (not shown). Values mean ± SD. *p < .05 vs. ECCtrl. ns—Not significant. On‐TARGETplus human RUNX1 siRNA—Seq.#1: UGACAACCCUCUCUGCAGA; Seq#2: GAAACUAGAUGAUCAGACC; Seq#3: CGAUAGGUCUCACGCAACA. Seq#4: CAAAUGAUCUGGUGGUUAU. The ON‐TARGETplus Non‐targeting Control siRNA sequences used for Stub1 siRNA (Figure 3) served as negative controls. (C) Representative WB of lysates of ECCtrl, ECLPS and ECLPS exposed to MPs(d7), using Cdk6 and Ccnd2 antibodies.(D) Densitometry indicated that MPs(d7) exposure increases 1.4‐fold ECLPS proliferation. Actin served as loading control. The ratios Cdk6/Actin and Ccnd2/Actin are shown, as means ± SD. ***p < .0004 ECLPS vs. corresponding ECCtrl; *p < .02 and **P < .007 ECLPS+MPs(d7) vs. corresponding ECLPS. Values mean ± SD. *n* = 3 independent experiments using three different MPs preparations. ns—not significant.

Thus, MSCs‐derived MPs expressing RUNX*1^p66^* can translate into an effective and clinically safe cell‐free therapeutic approach to treat ARDS, a desirable alternative to avoid multiple administrations of allogeneic MSCs that have the potential to increase the risk of immunogenicity.^8^

## Supporting information

Supporting Information

**Figure S1** Characterisation of MSCs‐derived MPs—(A) Western Blot of RUNX1 expression in lysates of cultured bone marrow‐derived MSCs (d2–d8) as well in lysates of the MPs released in the growth media at day 7 (b1); 30  μg protein/lane. RUNX1 expression was investigated each day starting at about 5% confluence (day 2) and after the cultures had expanded to about 80% confluence (day 8). The expression of the RUNX1 isoform with Mr 52 kDa was detected starting at day 2 at each time point analysed, while RUNX1*^p66^* was transiently expressed (days 6 and 7) and released in the MPs recovered from the growth medium. *n* = 3 independent experiments. The isolated MPs were characterised according to the International Society for Extracellular Vesicles recent guidelines.^3^ (B–F) Flow cytometry analysis of MPs preparations labelled with fluorophore‐conjugated MSCs specific cell surface markers CD44‐APC‐eFluor780, CD73‐PE and CD90‐APC. (B) 1.45, .88 and .45 μm beads were run, to determine approximate sizing of the MPs relative to the beads. (C) Size distribution and concentration of MPs preparations measured by nanoparticle tracking analyses (NTA). (D–F) MPs’ preparations express predominantly the MSC specific cell surface markers. The results of data analysis are shown as average percent of total gated events (at least 10 000 events/sample) ± SEM. (G) Lysates of MPs (60 μg total protein/lane) were analysed by sodium dodecyl‐sulfate polyacrylamide gel electrophoresis (SDS‐PAGE) and Western blotting using specific antibodies against CD63, CD81 CD9 tetraspanins, followed by the appropriate reporter antibodies. (H, h1) High‐resolution negative staining electron microscopy (EM) of MPs shows the double membrane structure and their ability to fuse to each other (arrow). MPs subjected to negative staining EM were not prepared by the critical point drying procedure. (I) Representative transmission EM shows two small vesicular structures in the lumen of a blood vessel in the lung of the LPS‐injected mouse, 1 h post‐MPs administration. *n* = 3 MPs different preparations in three independent experiments. LPS, lipopolysaccharide; MP, microparticle; MSC, mesenchymal stem cell.

**FIGURE S2** MPs‐immunoreactive to RUNX1*^p66^* show greater efficacy in improving EC barrier dysfunction and stimulating ECs proliferation compared to the parental MSCs. (A–F) MPs(7) show greater efficacy in mitigating EC barrier dysfunction compared to the parental MSCs. Male and female EC_Ctrl_ (A), EC_LPS_ (1 μg/mL LPS, 6 h; B), EC_LPS_ exposed to 10 μg MPs(d7), immunoreactive to RUNX1*^p66^* (C), EC_LPS_ exposed to 10^5^/well MSCs (D), EC_LPS_ exposed to 10 μg MPs(d5; G) and EC_LPS_ exposed to 10 μg MPs(d8; F) were immunostained with VE‐cadherin/Alexa Fluor 594 reporter antibodies. LPS was continuously present in the growth culture media during the 30 h treatment. ECs exposed to MSCs were transferred gradually in MSCs growth media and kept for 24 h pre‐treatment. The intercellular gaps were identified (dashed white shapes) and their surface was quantified using the NIH ImageJ (G). *p < .03 [EC_LPS_ + MPs(d5, d8) vs. EC_LPS_)]; **p < .008 (EC_LPS_ + MSCs vs. EC_LPS:_ ****p < .0001 ([EC_LPS_ + MPs(d7) vs. EC_LPS_]. The averages of gap surface for EC‐male and EC‐female were used for statistical analyses. *n* = 3 different experiments performed in triplicates, using at least three different MPs’ preparations. AU—arbitrary units. The Student's *t‐*test (two‐tailed, unpaired *t*‐tests) was used to compare samples and their controls (GraphPad Prizm 8.2.1 software). (H–M) Representative immunofluorescent staining of EC_Ctrl_ (H), EC_LPS_ (I), EC_LPS_+MSCs (J), EC_LPS_+10μg MPs(d5; K) and EC_LPS_+10μg MPs(d7; L) and EC_LPS_+10μg MPs(d8; M) using Ki67/anti‐mouse IgG Alexa Flour 594 antibodies. Arrowheads indicate some Ki67‐positive ECs. (N) Quantification of Ki67‐positive ECs. *p < .01 EC_LPS_ vs. EC_Ctrl_; **p < .037 EC_LPS_+ MSCs vs. EC_Ctrl_; ***p < .0004 EC_LPS_+MPs(d7) vs. EC_LPS_. The average number of Ki67‐positive ECs per 50 high‐power fields of view was used for statistical analyses. *n* = 3 experiments performed in triplicate. Values mean ± SD.

**FIGURE S3** Lack of contact between an EC and an MSC during MSC transmigration across the EC monolayer. (A) Representative EM illustrates the proximity and the lack of contact between an EC and MSC during transmigration of an MSC across the EC monolayer (boxed area). A tight junction with interconnected strands formed by the tight junction proteins between two ECs as well as the fusion points known as “kissing points” are shown for comparison (a1). This type of the cell–cell interaction was never detected between the ECs and MSCs. (B) Lower magnification EM illustrates an MSC in the sub‐endothelial space and (C) an MSC intermingles between ECs and attaches to the surface of the Petri dish; the highly magnified panels, (c1, c2) illustrate the lack of contact between ECs and the MSC. The attachment to the surface of the Petri dish is strong enough, it cannot be washed out, and thus it may interfere with EdU^+^ assay and proliferative ECs count. To overcome this limitation, MSCs were labelled with PKH67 cell membrane stain, D, arrows, and their EdU^+^ nuclei when present, were excluded from counting. Bars: 100 nm (A); 150 nm (B). Bar: 20 μm.

**FIGURE S4** Hyperproliferative bronchial epithelium and fibroproliferation in the LPS‐injected mice. (A) A proliferative response of bronchial epithelium is detected in the lungs of the LPS‐injected mice (sub‐lethal LPS dose of 8 mg/kg). The bronchial epithelium appeared irregular, with hyperplastic, hyperchromatic cells, crowded and tightly packed. (B) The bronchial epithelium in a mouse not injected with LPS is shown for comparison. LPS‐injected mice were treated with equivalent doses of MPs (36 μg) or parental MSCs (2 × 10^5^). Hyperplasia of bronchial epithelial cells was still detected to a similar degree, in the lungs of LPS‐injected mice after MPs(d7), (C) and MSCs (not shown) treatment. (D) Collagen deposition in the large perivascular cuffs (arrows), and in the airways (arrowheads) of LPS‐injected mice. (E) MPs(d7) and (F) MSCs treatment minimally ameliorates collagen deposition in the lungs of LPS‐injected mice. (G) Irregular bronchial epithelium with crowded and tightly packed cells (arrowheads), is still present, post‐MPs(d7) treatment. (H) The hydroxyproline content, an index of collagen accumulation was not significantly altered by MPs(d7) or MSCs treatment. *n* = 6 mice (3 males/3 females in three independent experiments, with three different MP preparations.

**FIGURE S5** RUNX1 ubiquitination in ECs exposed to the proteasome inhibitor MG132. (A) RUNX1 ubiquitination and (B) accumulation of ubiquitinated proteins in ECs exposed to the proteasome inhibitor MG132 (50M). The glycosylated RUNX1*^p66^*, assumed to be a stable isoform, accumulates (A, asterisk). C. Densitometric quantification of ubiquitinated RUNX1. While RUNX1 ubiquitination is detectable even in the absence of MG132, in ECs exposed to MG132 the ubiquitinated RUNX1 levels are greater for all sizes of ubiquitin chains detected; *p < .016; *p < .02 vs. RUNX1 ubiquitination in the absence of MG132. Values mean ± SD. (D) Overexpression of a DDK‐tagged Stub1, the E3 ubiquitin ligase that promotes RUNX1 degradation, caused significant decrease in RUNX1 protein expression, like endogenous Stub1. Actin served as loading control. (E) Expression of Stub1 in EC_LPS_ as well as in EC_LPS_ treated with MPs. (E) Representative WB analysis of Stub1 expression in EC_LPS_, EC_LPS_ treated with MPs(d5), MPs(d7), MPs(d8) as well as in EC_Ctrl_, with no MPs exposure. Stub1 expression is increased by two‐fold in EC_LPS_ compared to EC_Ctrl_. MPs (d5) and MPs (d8) treatment reduced the LPS‐triggered upregulation of Stub1 to levels not significantly different from EC_Ctrl_. By contrast, EC_LPS_ treated with MPs(d7), which transfer the RUNX1*^p66^* to LPS‐injured ECs, still show 1.4‐fold increase in Stub1 expression; the observation is consistent with Stub1 involvement in the rapid ubiquitin‐mediated turnover of Runx1*^p66^*, a common regulatory mechanism for the transcription factors involved in cell‐cycle control, such as RUNX1*^p66^*. ***p < .0004 EC_LPS_+MP(d5) vs. EC_LPS_ and EC_Ctrl_ vs. EC_LPS_. **p < .006 EC_LPS_+MP(d7) vs. EC_LPS_; **p < .0058 EC_LPS_+MP(d8) vs. EC_LPS_; **p < .009 EC_LPS_+MP(d7) vs. EC_LPS_+MPs(d5); ns—not significant. Actin was used as loading control. *n* = 3 independent experiments.
