# Supplementary material for: RUNX1 mediates the therapeutic effects of mesenchymal stem cells‐derived microparticles in acute respiratory distress syndrome
Source: Clin Transl Med. 2023 Nov 13;13(11):e1455. doi: 10.1002/ctm2.1455 (PMC10641787; doi:10.1002/ctm2.1455)
Supplement: Supplementary file 1 — Supporting Information [file CTM2-13-e1455-s003.docx]

**Figure S1. Characterization of MSCs-derived MPs –** **A.** Western Blot of RUNX1 expression in lysates of cultured bone marrow-derived MSCs (d2 – d8) as well in lysates of the MPs released in the growth media at day 7 (**b1**); 30 μg protein/lane. RUNX1 expression was investigated each day starting at about 5% confluence (day 2) and after the cultures had expanded to about 80% confluence (day 8). The expression of the RUNX1 isoform with Mr 52 kDa was detected starting at day 2 at each time point analyzed, while RUNX1*^p66^* was transiently expressed (days 6 and 7) and released in the MPs recovered from the growth medium. n= 3 independent experiments. The isolated MPs were characterized according to the International Society for Extracellular Vesicles recent guidelines^3^. **B-F.** Flow cytometry analysis of MPs preparations labelled with fluorophore-conjugated MSCs specific cell surface markers CD44-APC-eFluor780, CD73-PE and CD90-APC. **B.** 1.45µm, 0.88µm and 0.45µm beads were run, to determine approximate sizing of the MPs relative to the beads. **C.** Size distribution and concentration of MPs preparations measured by nanoparticle tracking analyses (NTA). **D, E, F.** MPs preparations express predominantly the MSCs specific cell surface markers. The results of data analysis are shown as average % of total gated events (at least 10,000 events/sample) ± SEM.

**G.** Lysates of MPs (60 μg total protein/lane) were analyzed by SDS PAGE and Western blotting using specific antibodies against CD63, CD81 CD9 tetraspanins, followed by the appropriate reporter antibodies. **H, h1.** High-resolution negative staining electron microscopy (EM) of MPs shows the double membrane structure and their ability to fuse to each other (arrow). MPs subjected to negative staining EM were not prepared by the critical point drying procedure. **I**. Representative transmission EM shows two small vesicular structures in the lumen of a blood vessel in the lung of the LPS-injected mouse, 1h post-MPs administration. n=3 MPs different preparations in 3 independent experiments.

**Figure S2. MPs-immunoreactive to RUNX1*^p66^* show greater efficacy in improving EC barrier dysfunction and stimulating ECs proliferation compared to the parental MSCs. A – F.** MPs(7) show greater efficacy in mitigating EC barrier dysfunction compared to the parental MSCs. Male and female EC_Ctrl_ (**A**), EC_LPS_ (1µg/ml LPS, 6h; **B**), EC_LPS_ exposed to 10µg MPs(d7), immunoreactive to RUNX1*^p66^* (**C**), EC_LPS_ exposed to 10^5^/well MSCs (**D**), EC_LPS_ exposed to 10µg MPs(d5; **G**) and EC_LPS_ exposed to 10µg MPs(d8; **F)** were immunostained with VE-cadherin/Alexa Fluor 594 reporter antibodies. LPS was continuously present in growth culture media during the 30h treatment. ECs exposed to MSCs were transferred gradually in MSCs growth media and kept for 24h pre-treatment. The intercellular gaps were identified (dashed white shapes) and their surface was quantified using the NIH ImageJ (**G)**. *p<0.03 [EC_LPS_ + MPs(d5, d8) vs EC_LPS_)]; **p<0.008 (EC_LPS_ + MSCs vs EC_LPS:_ **** <0.0001 ([EC_LPS_ + MPs(d7) vs EC_LPS_]. The averages of gap surface for EC-Male and EC-Female were used for statistical analyses. n=3 different experiments performed in triplicates, using at least 3 different MPs preparations. AU - arbitrary units. The student’s *t-*test (two tailed, unpaired *t*-tests) was used to compare samples and their controls (GraphPad Prizm 8.2.1 software).

**H-M**. Representative immunofluorescent staining of EC_Ctrl_ (**H**), EC_LPS_ **(I**), EC_LPS_+MSCs (**J**), EC_LPS_+10μg MPs(d5; **K**) and EC_LPS_+10μg MPs(d7; **L**) and EC_LPS_+10μg MPs(d8; **M**) using Ki67/anti-mouse IgG Alexa Flour 594 antibodies. Arrowheads indicate some Ki67-positive ECs. **N.** Quantification of Ki67-positive ECs. *p<0.01 EC_LPS_ vs EC_Ctrl_; **p<0.037 EC_LPS_+ MSCs vs EC_Ctrl_; ***p<0.0004 EC_LPS_+MPs(d7) vs EC_LPS_. The average number of Ki67-positive ECs per 50 high-power fields of view were used for statistical analyses. n=3 experiments performed in triplicate. Values mean ± SD.

**FIGURE S3.** **Lack of contact between an EC and a MSC during MSC transmigration across the EC monolayer.** **A.** Representative EM illustrates the proximity and the lack of contact between an EC and MSC during transmigration of a MSC across the EC monolayer (boxed area). A tight junction with interconnected strands formed by the tight junction proteins between two ECs as well as the fusion points known as “kissing points” are shown for comparison (**a1)** This type of cell-cell interaction was never detected between the ECs and MSCs. **B.** Lower magnification EM illustrates a MSC in the sub-endothelial space and **C**. a MSC intermingles between ECs and attaches to the surface of the Petri dish; the highly magnified panels, **c1, c2**, illustrate the lack of contact between ECs and the MSC. The attachment to the surface of the Petri dish is strong enough, it cannot be washed out, and thus it may interfere with EdU^+^ assay and proliferative ECs count. To overcome this limitation, MSCs were labeled with PKH67 cell membrane stain, **Fig D**, arrows, and their EdU^+^ nuclei when present, were excluded from counting. Bars: 100 nm (A); 150 nm (B). Bar: 20µm.

**FIGURE S4. Hyperproliferative bronchial epithelium and fibroproliferation in the LPS-injected mice. A.** A proliferative response of bronchial epithelium is detected in the lungs of the LPS-injected mice (sub-lethal LPS dose of 8 mg/kg)**.** The bronchial epithelium appeared irregular, with hyperplastic, hyperchromatic cells, crowded and tightly packed. **B.** The bronchial epithelium in a mouse not injected with LPS is show for comparison. LPS-injected mice were treated with equivalent doses of MPs (36 µg) or parental MSCs (2x10^5^). Hyperplasia of bronchial epithelial cells was still detected to a similar degree, in the lungs of LPS-injected mice after MPs(d7), **Fig. 4C** and MSCs (not shown) treatment.  **D.** Collagen deposition in the large perivascular cuffs (arrows), and in the airways (arrowheads) of LPS-injected mice. **E.** MPs(d7) and **F.** MSCs treatment minimally ameliorates collagen deposition in the lungs of LPS-injected mice. **G.** Irregular bronchial epithelium with crowded and tightly packed cells (arrowheads), is still present, post-MPs(d7) treatment. **H.** The hydroxyproline content, an index of collagen accumulation was not significantly altered by MPs(d7) or MSCs treatment. n=6 mice (3 males/3 females in 3 independent experiments, with 3 different MPs preparations.

**FIGURE S5.**  **RUNX1 ubiquitination in ECs exposed to the proteasome inhibitor MG132.**

**A.** RUNX1 ubiquitination and **B**. accumulation of ubiquitinated proteins in ECs exposed to the proteasome inhibitor MG132 (50μM)**.** The glycosylated RUNX1*^p66^,* assumed to be a stable isoform, accumulates (**A,** asterisk). **C.** Densitometric quantification of ubiquitinated RUNX1. While RUNX1 ubiquitination is detectable even in the absence of MG132, in ECs exposed to MG132 the ubiquitinated RUNX1 levels are greater for all sizes of ubiquitin chains detected; *p<0.016; *p<0.02 vs. RUNX1 ubiquitination in the absence of MG132. Values mean ± SD. **D.** Overexpression of a DDK-tagged Stub1, the E3 ubiquitin ligase that promotes RUNX1 degradation, caused significant decrease in RUNX1 protein expression, like endogenous Stub1**.**  Actin served as loading control. E. Expression of Stub1 in EC_LPS_ as well as in EC_LPS_ treated with MPs.

**E.** Representative WB analysis of Stub1 expression in EC_LPS_, EC_LPS_ treated with MPs(d5), MPs(d7), MPs(d8) as well as in EC_Ctrl_, with no MPs exposure. Stub1 expression is increased by 2-fold in EC_LPS_ compared to EC_Ctrl_. MPs (d5) and MPs (d8) treatment reduced the LPS-triggered upregulation of Stub1 to levels not significantly different from EC_Ctrl_. By contrast, EC_LPS_ treated with MPs(d7), which transfer the RUNX1*^p66^* to LPS-injured ECs, still show 1.4-fold increase in Stub1 expression; the observation is consistent with Stub1 involvement in the rapid ubiquitin-mediated turnover of Runx1*^p66^*, a common regulatory mechanism for the transcription factors involved in cell cycle control, such as RUNX1*^p66^*. ***p<0.0004 EC_LPS_+MP(d5) vs EC_LPS_ and EC_Ctrl_ vs EC_LPS_. **p<0.006 EC_LPS_+MP(d7) vs EC_LPS_; **p<0.0058 EC_LPS_+MP(d8) vs EC_LPS_; **p<0.009 EC_LPS_+MP(d7) vs EC_LPS_+MPs(d5); ns – not significant. Actin was used as loading control. n=3 independent experiments.
