# Supplementary figures and images for: RUNX1 mediates the therapeutic effects of mesenchymal stem cells‐derived microparticles in acute respiratory distress syndrome
Source: Clin Transl Med. 2023 Nov 13;13(11):e1455. doi: 10.1002/ctm2.1455 (PMC10641787; doi:10.1002/ctm2.1455)

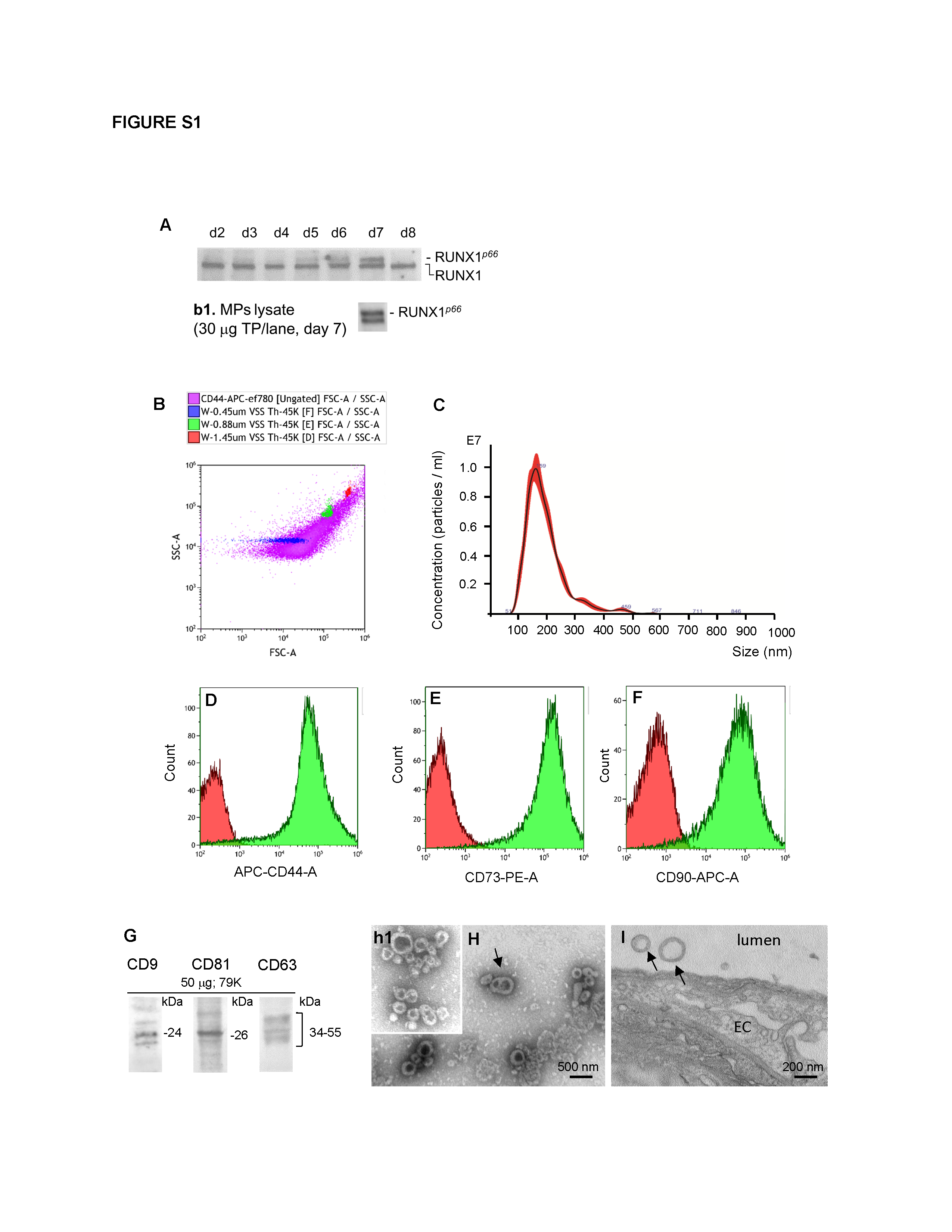

Supplement: Supplementary file 2 — Figure S1 Characterisation of MSCs‐derived MPs—(A) Western Blot of RUNX1 expression in lysates of cultured bone marrow‐derived MSCs (d2–d8) as well in lysates of the MPs released in the growth media at day 7 (b1); 30 μg protein/lane. RUNX1 expression was investigated each day starting at about 5% confluence (day 2) and after the cultures had expanded to about 80% confluence (day 8). The expression of the RUNX1 isoform with Mr 52 kDa was detected starting at day 2 at each time point analysed, while RUNX1 p66 was transiently expressed (days 6 and 7) and released in the MPs recovered from the growth medium. n = 3 independent experiments. The isolated MPs were characterised according to the International Society for Extracellular Vesicles recent guidelines. 3 (B–F) Flow cytometry analysis of MPs preparations labelled with fluorophore‐conjugated MSCs specific cell surface markers CD44‐APC‐eFluor780, CD73‐PE and CD90‐APC. (B) 1.45, .88 and .45 μm beads were run, to determine approximate sizing of the MPs relative to the beads. (C) Size distribution and concentration of MPs preparations measured by nanoparticle tracking analyses (NTA). (D–F) MPs’ preparations express predominantly the MSC specific cell surface markers. The results of data analysis are shown as average percent of total gated events (at least 10 000 events/sample) ± SEM. (G) Lysates of MPs (60 μg total protein/lane) were analysed by sodium dodecyl‐sulfate polyacrylamide gel electrophoresis (SDS‐PAGE) and Western blotting using specific antibodies against CD63, CD81 CD9 tetraspanins, followed by the appropriate reporter antibodies. (H, h1) High‐resolution negative staining electron microscopy (EM) of MPs shows the double membrane structure and their ability to fuse to each other (arrow). MPs subjected to negative staining EM were not prepared by the critical point drying procedure. (I) Representative transmission EM shows two small vesicular structures in the lumen of a blood vessel in the lung of the LPS‐inj [file CTM2-13-e1455-s006.tiff]

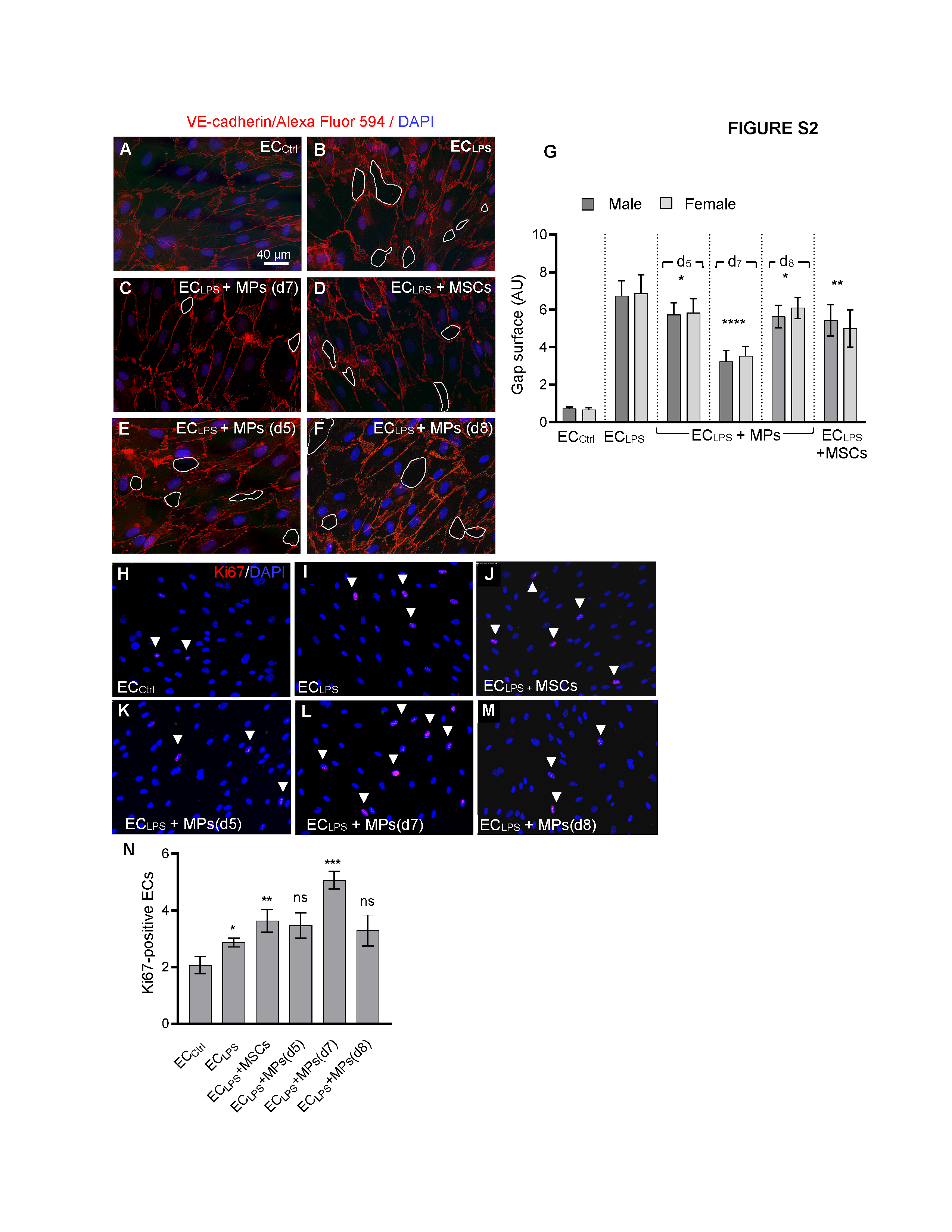

Supplement: Supplementary file 3 — FIGURE S2 MPs‐immunoreactive to RUNX1 p66 show greater efficacy in improving EC barrier dysfunction and stimulating ECs proliferation compared to the parental MSCs. (A–F) MPs(7) show greater efficacy in mitigating EC barrier dysfunction compared to the parental MSCs. Male and female ECCtrl (A), ECLPS (1 μg/mL LPS, 6 h; B), ECLPS exposed to 10 μg MPs(d7), immunoreactive to RUNX1 p66 (C), ECLPS exposed to 105/well MSCs (D), ECLPS exposed to 10 μg MPs(d5; G) and ECLPS exposed to 10 μg MPs(d8; F) were immunostained with VE‐cadherin/Alexa Fluor 594 reporter antibodies. LPS was continuously present in the growth culture media during the 30 h treatment. ECs exposed to MSCs were transferred gradually in MSCs growth media and kept for 24 h pre‐treatment. The intercellular gaps were identified (dashed white shapes) and their surface was quantified using the NIH ImageJ (G). *p < .03 [ECLPS + MPs(d5, d8) vs. ECLPS)]; **p < .008 (ECLPS + MSCs vs. ECLPS: ****p < .0001 ([ECLPS + MPs(d7) vs. ECLPS]. The averages of gap surface for EC‐male and EC‐female were used for statistical analyses. n = 3 different experiments performed in triplicates, using at least three different MPs’ preparations. AU—arbitrary units. The Student's t‐test (two‐tailed, unpaired t‐tests) was used to compare samples and their controls (GraphPad Prizm 8.2.1 software). (H–M) Representative immunofluorescent staining of ECCtrl (H), ECLPS (I), ECLPS+MSCs (J), ECLPS+10μg MPs(d5; K) and ECLPS+10μg MPs(d7; L) and ECLPS+10μg MPs(d8; M) using Ki67/anti‐mouse IgG Alexa Flour 594 antibodies. Arrowheads indicate some Ki67‐positive ECs. (N) Quantification of Ki67‐positive ECs. *p < .01 ECLPS vs. ECCtrl; **p < .037 ECLPS+ MSCs vs. ECCtrl; ***p < .0004 ECLPS+MPs(d7) vs. ECLPS. The average number of Ki67‐positive ECs per 50 high‐power fields of view was used for statistical analyses. n = 3 experiments performed in triplicate. Values mean ± SD. [file CTM2-13-e1455-s005.tiff]

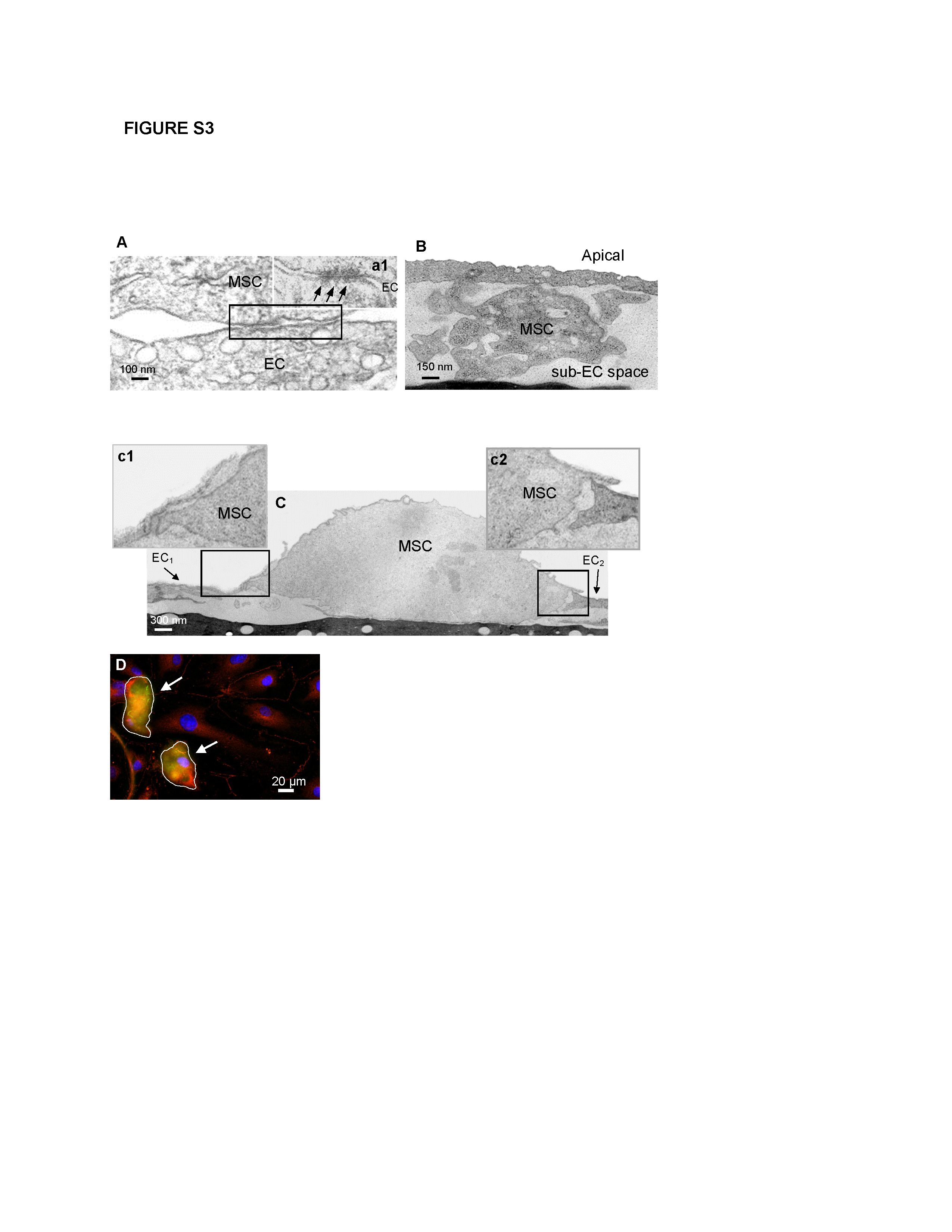

Supplement: Supplementary file 4 — FIGURE S3 Lack of contact between an EC and an MSC during MSC transmigration across the EC monolayer. (A) Representative EM illustrates the proximity and the lack of contact between an EC and MSC during transmigration of an MSC across the EC monolayer (boxed area). A tight junction with interconnected strands formed by the tight junction proteins between two ECs as well as the fusion points known as “kissing points” are shown for comparison (a1). This type of the cell–cell interaction was never detected between the ECs and MSCs. (B) Lower magnification EM illustrates an MSC in the sub‐endothelial space and (C) an MSC intermingles between ECs and attaches to the surface of the Petri dish; the highly magnified panels, (c1, c2) illustrate the lack of contact between ECs and the MSC. The attachment to the surface of the Petri dish is strong enough, it cannot be washed out, and thus it may interfere with EdU+ assay and proliferative ECs count. To overcome this limitation, MSCs were labelled with PKH67 cell membrane stain, D, arrows, and their EdU+ nuclei when present, were excluded from counting. Bars: 100 nm (A); 150 nm (B). Bar: 20 μm. [file CTM2-13-e1455-s004.tiff]

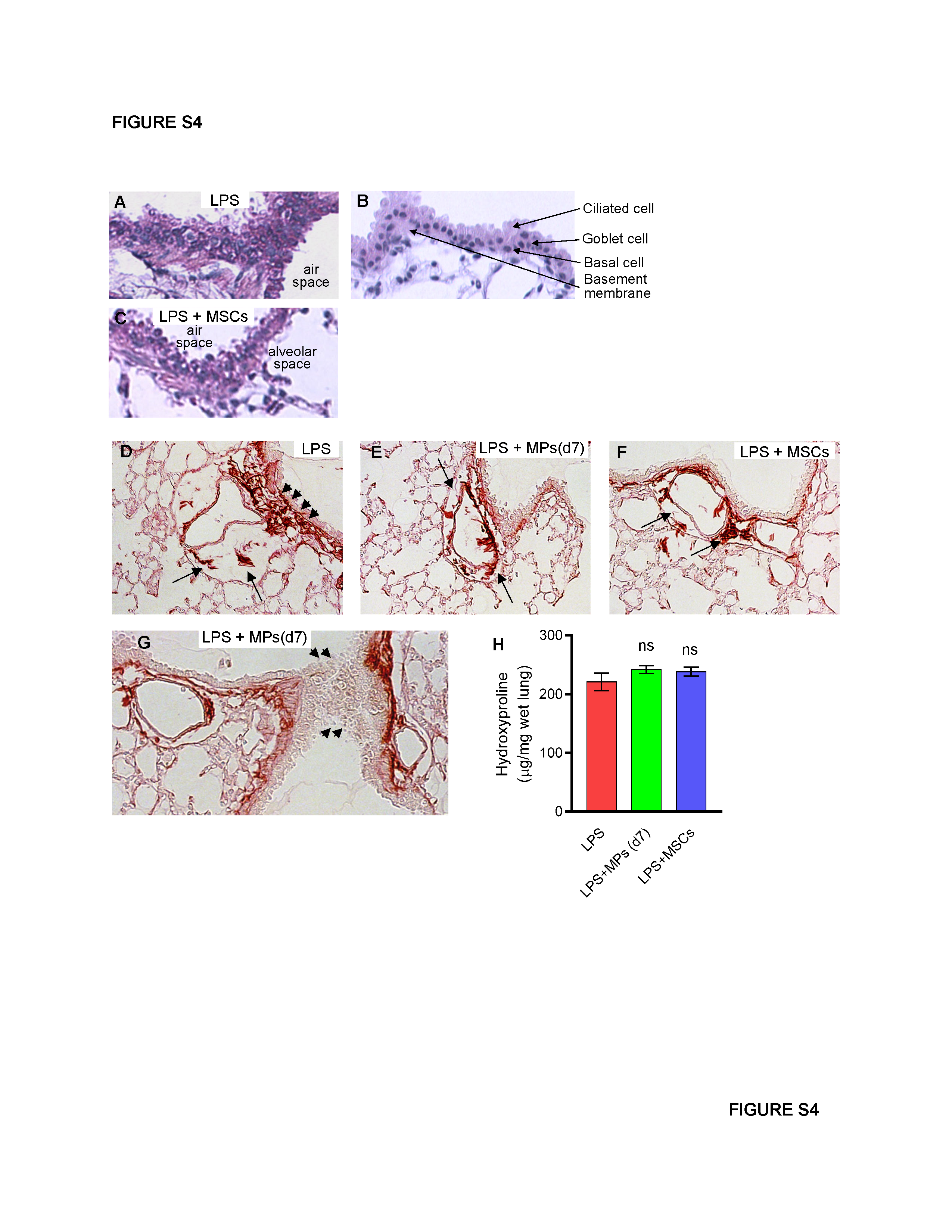

Supplement: Supplementary file 5 — FIGURE S4 Hyperproliferative bronchial epithelium and fibroproliferation in the LPS‐injected mice. (A) A proliferative response of bronchial epithelium is detected in the lungs of the LPS‐injected mice (sub‐lethal LPS dose of 8 mg/kg). The bronchial epithelium appeared irregular, with hyperplastic, hyperchromatic cells, crowded and tightly packed. (B) The bronchial epithelium in a mouse not injected with LPS is shown for comparison. LPS‐injected mice were treated with equivalent doses of MPs (36 μg) or parental MSCs (2 × 10^5^). Hyperplasia of bronchial epithelial cells was still detected to a similar degree, in the lungs of LPS‐injected mice after MPs(d7), (C) and MSCs (not shown) treatment. (D) Collagen deposition in the large perivascular cuffs (arrows), and in the airways (arrowheads) of LPS‐injected mice. (E) MPs(d7) and (F) MSCs treatment minimally ameliorates collagen deposition in the lungs of LPS‐injected mice. (G) Irregular bronchial epithelium with crowded and tightly packed cells (arrowheads), is still present, post‐MPs(d7) treatment. (H) The hydroxyproline content, an index of collagen accumulation was not significantly altered by MPs(d7) or MSCs treatment. n = 6 mice (3 males/3 females in three independent experiments, with three different MP preparations. [file CTM2-13-e1455-s002.tiff]

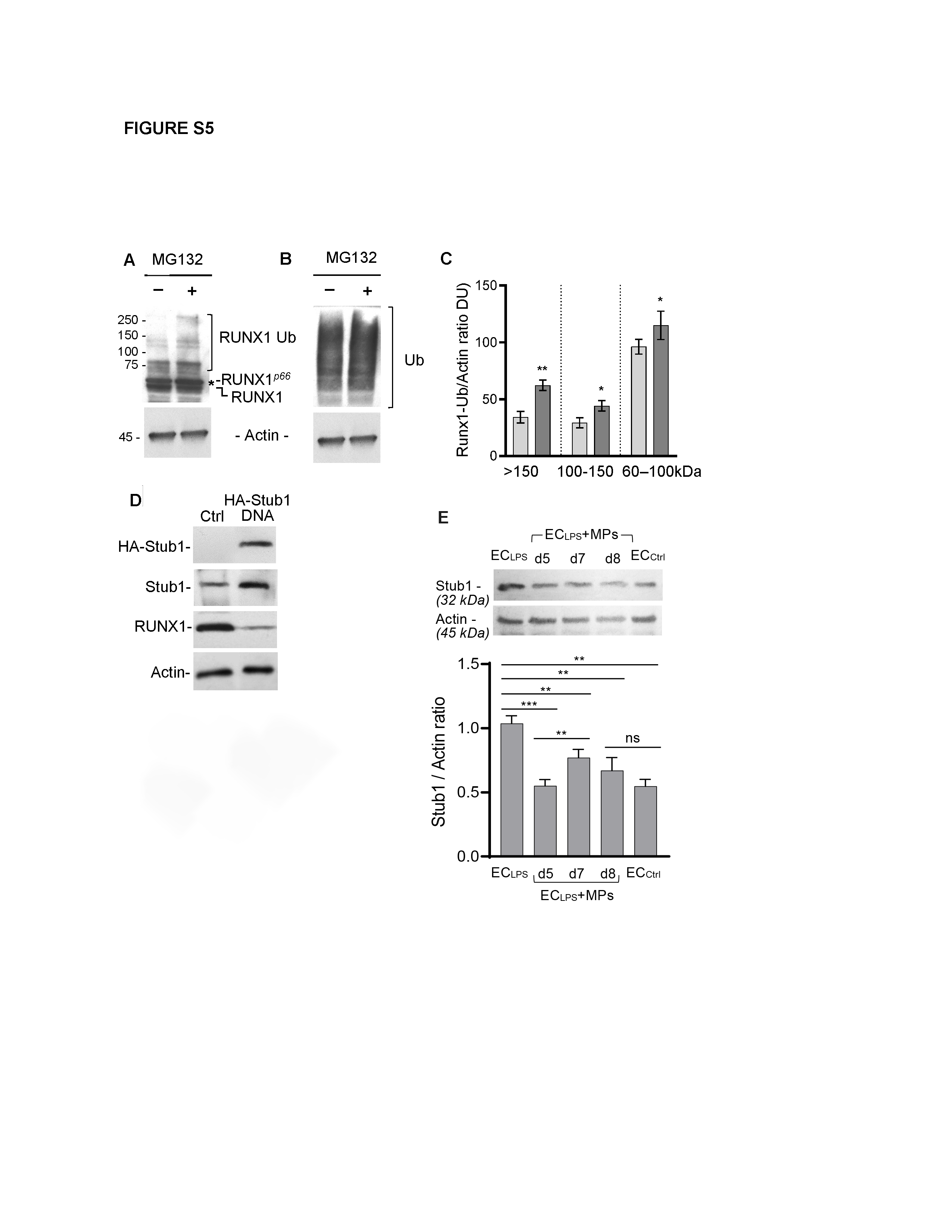

Supplement: Supplementary file 6 — FIGURE S5 RUNX1 ubiquitination in ECs exposed to the proteasome inhibitor MG132. (A) RUNX1 ubiquitination and (B) accumulation of ubiquitinated proteins in ECs exposed to the proteasome inhibitor MG132 (50M). The glycosylated RUNX1 p66 , assumed to be a stable isoform, accumulates (A, asterisk). C. Densitometric quantification of ubiquitinated RUNX1. While RUNX1 ubiquitination is detectable even in the absence of MG132, in ECs exposed to MG132 the ubiquitinated RUNX1 levels are greater for all sizes of ubiquitin chains detected; *p < .016; *p < .02 vs. RUNX1 ubiquitination in the absence of MG132. Values mean ± SD. (D) Overexpression of a DDK‐tagged Stub1, the E3 ubiquitin ligase that promotes RUNX1 degradation, caused significant decrease in RUNX1 protein expression, like endogenous Stub1. Actin served as loading control. (E) Expression of Stub1 in ECLPS as well as in ECLPS treated with MPs. (E) Representative WB analysis of Stub1 expression in ECLPS, ECLPS treated with MPs(d5), MPs(d7), MPs(d8) as well as in ECCtrl, with no MPs exposure. Stub1 expression is increased by two‐fold in ECLPS compared to ECCtrl. MPs (d5) and MPs (d8) treatment reduced the LPS‐triggered upregulation of Stub1 to levels not significantly different from ECCtrl. By contrast, ECLPS treated with MPs(d7), which transfer the RUNX1 p66 to LPS‐injured ECs, still show 1.4‐fold increase in Stub1 expression; the observation is consistent with Stub1 involvement in the rapid ubiquitin‐mediated turnover of Runx1 p66 , a common regulatory mechanism for the transcription factors involved in cell‐cycle control, such as RUNX1 p66 . ***p < .0004 ECLPS+MP(d5) vs. ECLPS and ECCtrl vs. ECLPS. **p < .006 ECLPS+MP(d7) vs. ECLPS; **p < .0058 ECLPS+MP(d8) vs. ECLPS; **p < .009 ECLPS+MP(d7) vs. ECLPS+MPs(d5); ns—not significant. Actin was used as loading control. n = 3 independent experiments. [file CTM2-13-e1455-s001.tiff]
